# Disruption of Central Antioxidant Property of Nuclear Factor Erythroid 2-Related Factor 2 Worsens Circulatory Homeostasis with Baroreflex Dysfunction in Heart Failure

**DOI:** 10.3390/ijms19030646

**Published:** 2018-02-25

**Authors:** Takuya Kishi

**Affiliations:** Department of Advanced Risk Stratification for Cardiovascular Diseases, Center for Disruptive Cardiovascular Medicine, Kyushu University, 3-1-1 Maidashi, Higashi-Ku, Fukuoka 812-8582, Japan; tkishi@cardiol.med.kyushu-u.ac.jp; Tel.: +81-92-642-6428; Fax: +81-92-642-5374

**Keywords:** heart failure, brain, oxidative stress, homeostasis, baroreflex

## Abstract

Heart failure is defined as a disruption of circulatory homeostasis. We have demonstrated that baroreflex dysfunction strikingly disrupts circulatory homeostasis. Moreover, previous many reports have suggested that central excess oxidative stress causes sympathoexcitation in heart failure. However, the central mechanisms of baroreflex dysfunction with oxidative stress has not been fully clarified. Our hypothesis was that the impairment of central antioxidant property would worsen circulatory homeostasis with baroreflex dysfunction in heart failure. As the major antioxidant property in the brain, we focused on nuclear factor erythroid 2-related factor 2 (Nrf2; cytoprotective transcription factor). Hemodynamic and baroreflex function in conscious state were assessed by the radio-telemetry system. In the heart failure treated with intracerebroventricular (ICV) infusion of angiotensin II type 1 receptor blocker (ARB), sympathetic activation and brain oxidative stress were significantly lower, and baroreflex sensitivity and volume tolerance were significantly higher than in heart failure treated with vehicle. ICV infusion of Nrf2 activator decreased sympathetic activation and brain oxidative stress, and increased baroreflex sensitivity and volume tolerance to a greater extent than ARB. In conclusion, the disruption of central antioxidant property of Nrf2 worsened circulatory homeostasis with baroreflex dysfunction in heart failure.

## 1. Introduction

Abnormal and excessive sympathoexcitation is closely associated with the pathogenesis of heart failure [1,2,3]. The sympathetic nervous system is regulated by rostral ventrolateral medulla (RVLM) in brainstem of the central nervous system [4]. Central oxidative stress causes sympathoexcitation in rats with heart failure, and the oxidative stress being mainly produced by central angiotensin II type 1 receptor (AT_1_R) [5,6,7]. Recently, we have demonstrated that baroreflex failure induces striking volume intolerance in the absence of left ventricular (LV) dysfunction, and may play an important role in the pathogenesis of heart failure [8]. Patients with decompensated heart failure are supersensitive to volume overload, and a noticeable increase in left atrial pressure occurs rapidly. In contrast, normal subjects without heart failure have robust tolerance against volume infusion. These clinical features suggest that the dynamic robust volume tolerance might be an indicator of “circulatory homeostasis”, and that the disruption of which is equivalent to heart failure [8,9,10]. Cardiac output is determined by preload (stressed blood volume) and cardiac contractility [2,3], and baroreflex has been established to be a major and powerful regulator of both preload and cardiac contractility [1,2,3,9,10]. Baroreceptors are stretch receptors that are located within the arterial wall of elastic vessels, such as the aortic arch and carotid sinuses. The baroreflex is impaired in heart failure, with both reduced and preserved LV ejection fraction (LVEF) [3]. However, the central mechanisms of baroreflex dysfunction upon oxidative stress is yet to be fully clarified. 

Recently, nuclear factor erythroid 2-related factor 2 (Nrf2) has been in focus as an antioxidant and detoxification enzymes [11]. Nrf2-mediated neuroprotection is primarily conferred by astrocytes [12,13], and selective overexpression of Nrf2 under an astrocyte-specific promoter leads to the increased survival in mouse models of neurodegenerative diseases [14,15]. In these aspects, we recently showed that astrocyte-specific deletion of AT_1_R caused sympathoinhibition with the improvement of survival in mice with myocardial infarction (MI)-induced heart failure [16]. 

On the basis of the above information, we hypothesized that the impairment of central antioxidant property with Nrf2 could be associated with baroreflex dysfunction in heart failure. To validate the hypothesis, we examined hemodynamics, autonomic function, and baroreflex function in rats with myocardial infarction (MI)-induced heart failure, by coronary ligation, and compared to sham rats. We divided MI-induced heart failure into three groups: MI-induced heart failure treated with intracerebroventricular (ICV) infusion of AT_1_R blocker (ARB, losartan) (HF-ARB, *n* = 5), or with ICV infusion of Nrf2 activator, *tert*-butylhydroquinone (tBHQ) (HF-tBHQ, *n* = 5), or simply with ICV infusion of vehicle (HF-VEH, *n* = 5). Sham rats were also divided into three groups: treated with ICV infusion of ARB (Sham-ARB, *n* = 5), or with ICV infusion of tBHQ (Sham-tBHQ, *n* = 5), or simply with ICV infusion of vehicle (Sham-VEH, *n* = 5). 

## 2. Results

### 2.1. Assessment of Myocardial Infarction and Heart Failure 

One week after coronary ligation, LVEF and LV diastolic dimension were similar between in HF-VEH, HF-ARB, and HF-tBHQ (LVEF: 41.9 ± 2.5%, 42.3 ± 1.9%, and 41.5 ± 1.6%, *n* = 5 for each; LV diastolic dimension: 9.6 ± 0.4, 9.9 ± 0.3, and 9.9 ± 0.2 mm, *n* = 5 for each). Moreover, at the end of the experiments, there was no rats with small MI size (<25%) at all.

### 2.2. Hemodynamics, Autonomic Function, and Baroreflex Sensitivity

**Figure 1:**
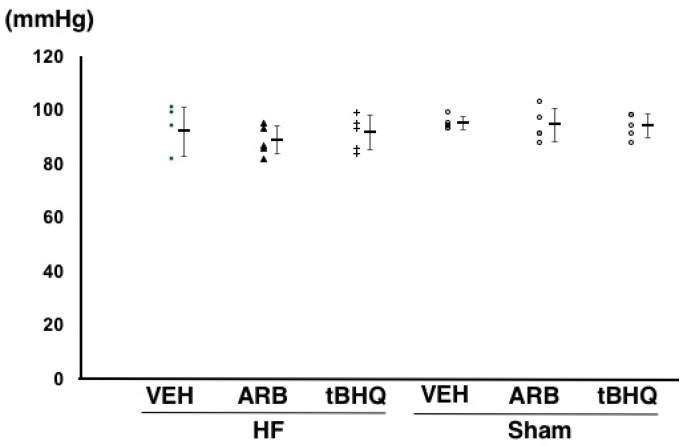
Mean arterial pressure of heart failure treated with intracerebroventricular (ICV) infusion of vehicle (HF-VEH), or with ICV infusion of angiotensin II type 1 receptor blocker (HF-ARB), or with ICV infusion of nuclear factor erythroid 2-related factor 2 activator, *tert*-butylhydroquinone (HF-tBHQ), or sham treated with ICV infusion of VEH, or with ICV infusion of ARB, or with ICV infusion of tBHQ. All data are shown in dot plot and mean ± standard deviation. *n* = 5 for each group.

**Figure 2:**
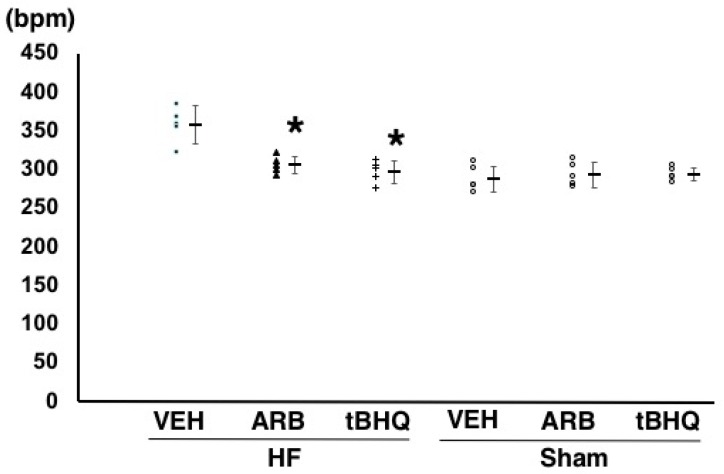
Heart rate of heart failure treated with intracerebroventricular (ICV) infusion of vehicle (HF-VEH), or with ICV infusion of angiotensin II type 1 receptor blocker (HF-ARB), or with ICV infusion of nuclear factor erythroid 2-related factor 2 activator, *tert*-butylhydroquinone (HF-tBHQ), or sham treated with ICV infusion of VEH, or with ICV infusion of ARB, or with ICV infusion of tBHQ. *n* = 5 for each group. All data are shown in dot plot and mean ± standard deviation. * *p* < 0.05 vs. HF-VEH in HF-ARB and HF-tBHQ. Bpm = beats per minute.

**Figure 3:**
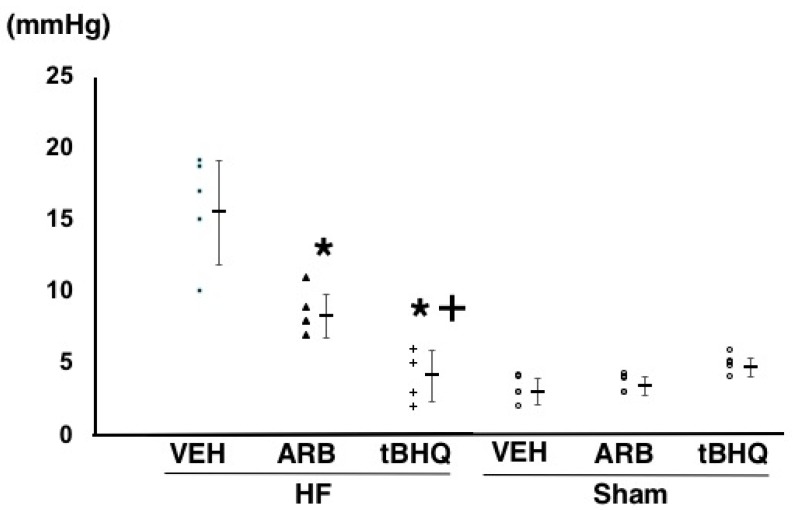
Left atrial pressure of heart failure treated with intracerebroventricular (ICV) infusion of vehicle (HF-VEH), or with ICV infusion of angiotensin II type 1 receptor blocker (HF-ARB), or with ICV infusion of nuclear factor erythroid 2-related factor 2 activator, *tert*-butylhydroquinone (HF-tBHQ), or sham treated with ICV infusion of VEH, or with ICV infusion of ARB, or with ICV infusion of tBHQ. *n* = 5 for each group. All data are shown in dot plot and mean ± standard deviation. * *p* < 0.05 vs. HF-VEH in HF-ARB and HF-tBHQ. ^+^ *p* < 0.05 vs. HF-ARB in HF-tBHQ.

At 14 days after the ICV infusion, mean arterial pressure was similar among HF-VEH, HF-ARB, and HF-tBHQ (92 ± 9, 89 ± 5, and 91 ± 7 mmHg, *n* = 5 for each) (Figure 1). However, heart rate were significantly higher in HF-VEH than in HF-ARB and HF-tBHQ (358 ± 24 vs. 305 ± 11 and 298 ± 15 bpm, *n* = 5 for each, *p* < 0.05) (Figure 2), and left atrial pressure was significantly lower in HF-tBHQ than in HF-VEH (5 ± 2 vs. 16 ± 4 mmHg, *n* = 5 for each, *p* < 0.05), and to a greater extent than in HF-ARB (8 ± 2 vs. 16 ± 4 mmHg, *n* = 5 for each, *p* < 0.05) (Figure 3). Mean arterial pressure was similar between in HF-VEH and Sham-VEH, and heart rate and left atrial pressure were significantly lower in Sham-VEH than in HF-VEH (Figure 1, Figure 2 and Figure 3). Mean arterial pressure, heart rate, and left atrial pressure were similar between in Sham-VEH, Sham-ARB, and Sham-tBHQ (Figure 1, Figure 2 and Figure 3). 

**Figure 4:**
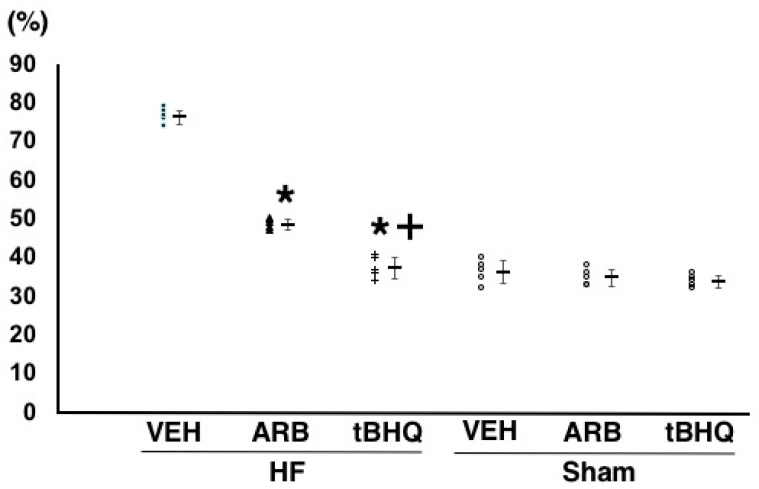
Low frequency component of the systolic arterial pressure variability of heart failure treated with intracerebroventricular (ICV) infusion of vehicle (HF-VEH), or with ICV imfusion of angiotensin II type 1 receptor blocker (HF-ARB), or with ICV infusion of nuclear factor erythroid 2-related factor 2 activator, *tert*-butylhydroquinone (HF-tBHQ), or sham treated with ICV infusion of VEH, or with ICV infusion of ARB, or with ICV infusion of tBHQ. *n* = 5 for each group. All data are shown in dot plot and mean ± standard deviation. * *p* < 0.05 vs. HF-VEH in HF-ARB and HF-tBHQ. ^+^ *p* < 0.05 vs. HF-ARB in HF-tBHQ.

As shown in Figure 4, the normalized unit of the low frequency component of the systolic arterial pressure variability, which is a parameter of sympathetic nerve activity, were significantly lower in HF-ARB than in HF-VEH (49 ± 1% vs. 76 ± 2%, *n* = 5 for each, *p* < 0.05 for each) and was significantly lower in HF-tBHQ than in HF-ARB (38 ± 3% vs. 49 ± 1%, *n* = 5 for each, *p* < 0.05 for each). The same parameter was significantly lower in Sham-VEH than in HF-VEH, and was similar between in Sham-VEH, Sham-ARB, and Sham-tBHQ (Figure 4).

**Figure 5:**
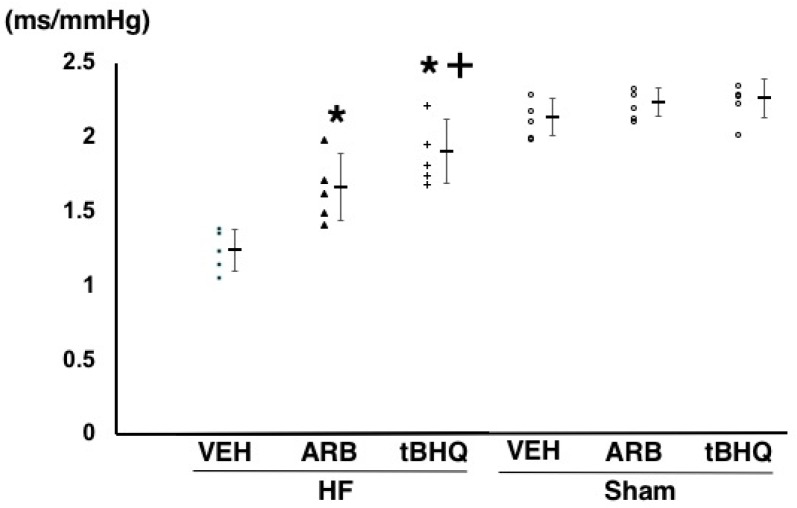
Baroreflex sensitivity of heart failure treated with intracerebroventricular (ICV) infusion of vehicle (HF-VEH), or with ICV infusion of angiotensin II type 1 receptor blocker (HF-ARB), or with ICV infusion of nuclear factor erythroid 2-related factor 2 activator, *tert*-butylhydroquinone (HF-tBHQ), or sham treated with ICV infusion of VEH, or with ICV infusion of ARB, or with ICV infusion of tBHQ. *n* = 5 for each group. All data are shown in dot plot and mean ± standard deviation. * *p* < 0.05 vs. HF-VEH in HF-ARB and HF-tBHQ. ^+^ *p* < 0.05 vs. HF-ARB in HF-tBHQ.

Baroreflex sensitivity was significantly higher in HF-tBHQ than in HF-VEH (1.87 ± 0.19 vs. 1.23 ± 0.16 ms/mmHg, *n* = 5 for each, *p* < 0.05), and to a greater extent than in HF-ARB (1.65 ± 0.21 vs. 1.23 ± 0.16 ms/mmHg, *n* = 5 for each, *p* < 0.05) (Figure 5). Baroreflex sensitivity was significantly higher in Sham-VEH than in HF-VEH, and was similar between in Sham-VEH, Sham-ARB, and Sham-tBHQ (Figure 5).

### 2.3. Oxidative Stress in the RVLM

**Figure 6:**
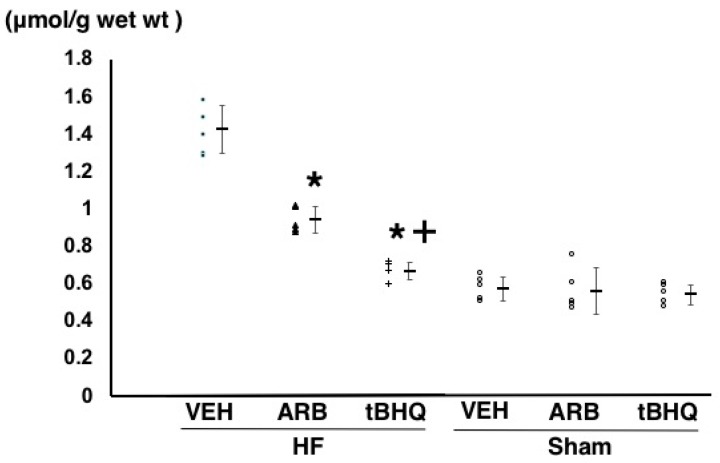
The amount of thiobarbituric acid-reactive substances in the rostral ventrolateral medulla of heart failure treated with intracerebroventricular (ICV) infusion of vehicle (HF-VEH), or with ICV infusion of angiotensin II type 1 receptor blocker (HF-ARB), or with ICV infusion of nuclear factor erythroid 2-related factor 2 activator, *tert*-butylhydroquinone (HF-tBHQ), or sham treated with ICV infusion of VEH, or with ICV infusion of ARB, or with ICV infusion of tBHQ. *n* = 5 for each group. All data are shown in dot plot and mean ± standard deviation. * *p* < 0.05 vs. HF-VEH in HF-ARB and HF-tBHQ. ^+^ *p* < 0.05 vs. HF-ARB in HF-tBHQ.

Oxidative stress in the RVLM assessed by thiobarbituric acid-reactive substances (TBARS) method were significantly lower in HF-ARB than in HF-VEH (0.95 ± 0.08 vs. 1.40 ± 0.10 μmol/g wet wt, *n* = 5 for each, *p* < 0.05), and further lower in HF-tBHQ than in HF-ARB (0.67 ± 0.04 vs. 0.95 ± 0.08 μmol/g wet wt, *n* = 5 for each, *p* < 0.05) (Figure 6). TBARS levels were significantly lower in Sham-VEH than in HF-VEH (0.59 ± 0.04 vs 1.40 ± 0.10 μmol/g wet wt, *n* = 5 for each, *p* < 0.05), and were similar between in Sham-VEH, Sham-ARB, and Sham-tBHQ (0.59 ± 0.04 vs. 0.56 ± 0.13 vs. 0.54 ± 0.08 μmol/g wet wt, *n* = 5 for each) (Figure 6).

### 2.4. Circulatory Homeostasis Assessed by Volume Tolerance

**Figure 7:**
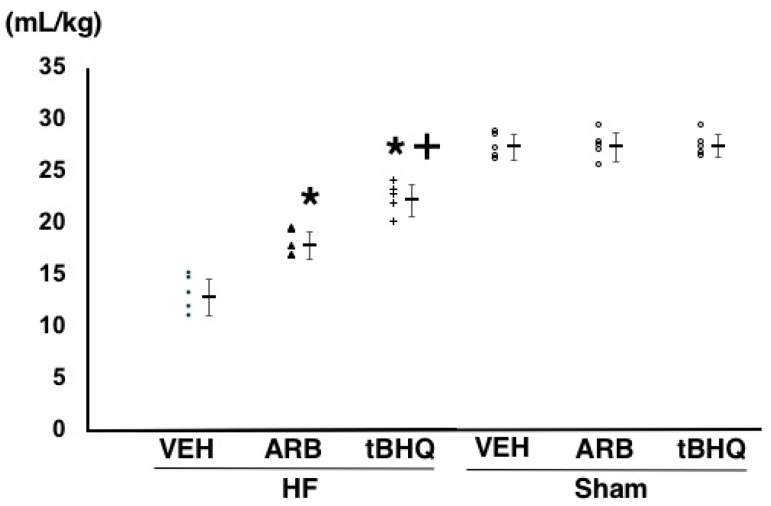
Infused volume at left atrial pressure to 14–16 mmHg of heart failure treated with intracerebroventricular (ICV) infusion of vehicle (HF-VEH), or with ICV infusion of angiotensin II type 1 receptor blocker (HF-ARB), or with ICV infusion of nuclear factor erythroid 2-related factor 2 activator, *tert*-butylhydroquinone (HF-tBHQ), or sham treated with ICV infusion of VEH, or with ICV infusion of ARB, or with ICV infusion of tBHQ. *n* = 5 for each group. All data are shown in dot plot and mean ± standard deviation. * *p* < 0.05 vs. HF-VEH in HF-ARB and HF-tBHQ. ^+^ *p* < 0.05 vs. HF-ARB in HF-tBHQ.

In HF-ARB and HF-tBHQ, volume loading by dextran infusion reproducibly increased left atrial pressure, but the changes were significantly smaller than in HF-VEH. Infused volume, with the elevation of left atrial pressure to 14–16 mmHg, was significantly larger in HF-ARB than in HF-VEH (18.1 ± 1.4 vs. 13.4 ± 1.9 mL/kg, *n* = 5 for each, *p* < 0.01), and further higher in HF-tBHQ than in HF-ARB (22.0 ± 1.6 vs. 18.1 ± 1.4 mL/kg, *n* = 5 for each, *p* < 0.01) (Figure 7). The same parameter was significantly higher in Sham-VEH than in HF-VEH, and was similar between in Sham-VEH, Sham-ARB, and Sham-tBHQ (Figure 7).

## 3. Discussion

The novel findings of the present study are as follows: (1) Oxidative stress in the RVLM was significantly lower in HF-tBHQ than in HF-VEH, and to a greater extent than in HF-ARB; (2) sympathoexcitation with baroreflex failure was attenuated in HF-tBHQ than in HF-VEH, and to a greater extent than in HF-ARB; and, (3) volume tolerance was improved in HF-tBHQ than in HF-VEH, and to a greater extent than in HF-ARB. These results suggest that disruption of central antioxidant property with Nrf2 worsened circulatory homeostasis with baroreflex dysfunction in heart failure. 

This is the first study to demonstrate that central antioxidant property strongly contributes to circulatory homeostasis with baroreflex. Heart failure is defined as a disruption of the dynamic circulatory homeostasis associated with interactions between multiple organs; brain integrates neural and neurohormonal information, from peripheral organs and controllers of peripheral organs, using autonomic nervous system [1,2,3]. It has been established that autonomic nervous system dysfunction, especially excess sympathoexcitation, worsens heart failure via the disruption of circulatory homeostasis [1,2,3,8,9,10]. The mechanisms that are involved in excess sympathoexcitation are baroreflex failure, attenuation of cardiopulmonary reflex modulation, cardiac sympathoexcitatory reflex related to increased cardiopulmonary filling pressure, sleep apnea, myocardial ischemia, obesity, and/or reflexes from exercising muscle [1,2,3]. Among them, most rapid and powerful regulator for sympathoexcitation is baroreflex control. The native arterial baroreceptor senses a change in arterial pressure and transmits the message to the vasomotor center via afferent nerves (the aortic depressor nerves and the carotid sinus nerves) [1,2,3]. The vasomotor center modulates the sympathetic outflow, depending on the inputs from the baroreceptors (central arc. of baroreflex). The efferent sympathetic nerve firing facilitates the cardiovascular actuators and induces resultant arterial pressure change (peripheral arc. of baroreflex). This negative feedback loop is the underlying biological mechanism that stabilizes arterial pressure. Previous studies have demonstrated that sympathoexcitation, with baroreflex failure is involved in the pathogenesis of heart failure [2,3]. Patients with heart failure are supersensitive to volume overload, and flash pulmonary edema often occurs transiently, which is rapidly resolved by intravascular volume reduction [3]. Recently, we demonstrated that baroreflex failure induces striking volume intolerance in the absence of LV dysfunction [8], and it that baroreflex modulates both cardiac function and vascular function to regulate arterial pressure; the baroreflex-induced changes in vascular resistance and stressed blood contribute to arterial pressure regulation to a far greater extent than the changes in contractility and heart rate [9]. These results strongly suggest that baroreflex failure could induce volume intolerance, independent of LV systolic function. In the present study, we demonstrated that central oxidative stress is closely associated with baroreflex dysfunction with sympathoexcitation in heart failure, and the antioxidant property of Nrf2 is the major factor for the excess oxidative stress in the RVLM.

We have demonstrated that central AT_1_R-induced oxidative stress causes sympathoexcitation in hypertension and heart failure. Moreover, baroreflex is found to have failed in hypertension and heart failure [5,6,7,17,18]. However, it has not been fully clarified whether the central AT_1_R-induced oxidative stress impaired baroreflex function or not. Considering these information from the literature, the present study introduced the new concept that AT_1_R-induced oxidative stress in the RVLM could have caused baroreflex failure and volume intolerance. Furthermore, these findings provide us a novel therapeutic target. Previously, we showed that telmisartan, one of the ARBs, improved the survival of rats with hypertensive heart failure, partially via the blockade of central AT_1_R [18]. Therefore, we consider that brain AT_1_R and antioxidant properties of Nrf2 would be the novel therapeutic target to improve circulatory homeostasis via amelioration of baroreflex and volume tolerance in heart failure.

Nrf2 is responsible for inducing gene expression of antioxidant enzymes and proteasome subunits, and the activation of the Nrf2 signaling is beneficial against neurodegeneration by lowering oxidative stress and eliminating the oxidatively damaged proteins. Nrf2 activity is repressed in neurons in vitro; only cultured astrocytes respond strongly to Nrf2 inducers, leading to the interpretation that Nrf2 signaling is largely restricted to astrocytes. However, Nrf2 activity can be observed in neurons from post-mortem brain tissue and animal models of diseases, and hence the above interpretation needed reconsideration [19]. Because our previous studies strongly suggested that AT_1_R in the astrocyte and oxidative stress were the major worsening factors in the pathophysiology and survival of heart failure [10,16], we focused on the Nrf2-mediated antioxidant properties in astrocytes. However, we did not assess the cell type-specific antioxidant properties associated with Nrf2 in the present study, and could not determine whether astrocyte-specific Nrf2 would be important in the circulatory homeostasis mediated with baroreflex or not. Further experiments with neuron- or astrocyte-specific deletion of Nrf2 would be required to address these aspects. 

To activate Nrf2 in the brain, we performed ICV infusion of tBHQ in the present study. Although we did not determine the changes in the activity and the expression of Nrf2 by tBHQ, sympathetic nerve activity was decreased with the reduction of oxidative stress in the RVLM and the improvement of baroreflex sensitivity, despite having similar arterial pressure. We have demonstrated that oxidative stress in the RVLM was the major factor activating sympathetic nerve activity [5,6,7,10]. The present results strongly suggest that the ICV infusion of tBHQ successfully activated Nrf2 in the RVLM and decreased oxidative stress with sympathoinhibition, thereby improving the baroreflex sensitivity. However, we could not conclude that the beneficial results were entirely caused by tBHQ, because the present methods were solely pharmacological interventions. To validate these further, cell-specific transgenic experiments with activation of Nrf2 would be necessary. 

Interestingly, in the present experiments, tBHQ did not alter either hemodynamic, baroreflex, oxidative stress in the RVLM, or circulatory homeostasis in sham rats. These results were similar to our previous studies with ARB, wherein ICV infusion of ARB did not alter oxidative stress in the brain and sympathetic nerve activity in hypertensive and metabolic rats [6,7]. Although we did not measure activation and the expression of Nrf2 in the present study, Nrf2 would not be activated and tBHQ could not activate Nrf2, in sham rats. In addition, we speculated that Nrf2 was not activated sufficiently in response to oxidative stress while tBHQ successfully activated Nrf2 in heart failure. We will plan further experiments to clarify why tBHQ could not activate Nrf2 in sham rats while did so successfully in case of heart failure. 

There are several limitations of the present study. First, we did not assess the expression of Nrf2 by immunohistochemical analysis or western blotting, whereas these two data could strengthen our results. Because of this insufficient aspect with only pharmacological and physiological methods, we could not entirely conclude that Nrf2 is the major key molecule of the central antioxidant property in the regulation of circulatory homeostasis. Second, we did not identify the area that was affected by ICV of ARB or tBHQ; we only assessed the vasomotor center of RVLM, not in other areas such as nucleus tractus solitarii, subfornical organ, and/or paraventricular nucleus. In the present study, we focused on sympathoexcitation and baroreflex, and we assessed them only in the vasomotor center. In the future experiments, other areas associated with sympathetic nervous system need to be examined. Finally, the sample sizes in each group were quite small (*n* = 5). Although the results were statistically significant, the present experiment could not prove the concept at all, because of the inadequate sample size [20]. Further assessments in larger sample sizes with cell-specific intervention of Nrf2 based on the present preliminary experiment should be done in future. 

## 4. Materials and Methods 

The Committee on the Ethics of Animal Experiments at the Kyushu University Graduate School of Medical Sciences reviewed and approved the present experiments (A28-193-0, approved at 12 April 2018). I have conducted the present experiments according to the Guidelines for Animal Experiments of Kyushu University. 

### 4.1. Animals and General Procedures 

14- to 16-week-old male Sprague-Dawley rats (Japan SLC Inc., Hamamatsu, Japan) weighing 450–650 g were induced for MI by ligating left coronary artery. I anesthetized rats with sodium pentobarbital (50 mg/kg intraperitoneally) with the mechanical ventilation. We ligated the left coronary artery with 5-0 silk. To select only the rats with established heart failure prior to initiate ICV infusion, among the survived rats at one week after coronary ligation, I excluded the rats defined by the prior exclusion criteria; (1) LV wall motion did not have asynergy, or LVEF was over 50%, or LV diastolic diameter was under 9.0 mm assessed by echocardiography (SSA-380A, TOSHIBA, Tokyo, Japan), (2) body weight did not increase when compared to that before coronary ligation, (3) 24-h averaged mean left atrial pressure was under 10 mmHg, (4) food and water intake were subjectively poor, and (5) activity was subjectively decreased, as in our previous study [21]. After the experimental protocols, the brain and heart of rats were removed with an over dose of pentobarbital, and the harvested heart fixed with 10% formaldehyde was sliced and stained with Masson’s trichrome stain to calculate infarct size. Rats with small MI size (<25%) was excluded from this study to select the rats with established heart failure.

To perform the ICV infusion, osmotic minipump (Alzet 2002; DURECT Corporation, Cupertino, CA) was implanted into the right lateral cerebral ventricle of the brain. Losartan (1 mg·kg^−1^·day^−1^) and tBHQ (1mM) in artificial cerebrospinal fluid or vehicle (only artificial cerebrospinal fluid) was infused at 0.5 µL/h for 14 days. I selected the present dose of losartan, because the ICV infusion of 1 mg·kg^−1^·day^−1^ losartan did not block peripheral AT_1_R, as in our previous study [22]. The present dose of tBHQ was determined according to a previous study [23].

### 4.2. Assessment of Hemodynamics, Autonomic Function, and Baroreflex Function 

We used a radio-telemetry system (Data Sciences International, St. Paul, MN, USA) to evaluate the arterial pressure, heart rate, and left atrial pressure, similar to our previous study [24]. Telemetry catheters were inserted into the abdominal aorta and left atrium prior to coronary ligation, and 24-h monitoring was initiated in the conscious states. Arterial pressure and left atrial pressure were separately recorded at a 500 Hz sampling frequency and averaged using 1-s bins. The power spectra for the systolic arterial pressure and heart rate were calculated by an adaptive autoregressive model, as in our previous study [9,24]. 

The sympathetic nerve activities were presented as the normalized unit of the low frequency component (0.04–0.60 Hz) of the systolic arterial pressure variability [6,7]. Baroreflex sensitivity was measured using the spontaneous sequence method, as in our previous study [6,7,17,18,22].

### 4.3. Measurement of Thiobarbituric Acid-Reactive Substances in the RVLM

To obtain the RVLM tissues, the rats were deeply anesthetized with sodium pentobarbital (100 mg/kg IP) and perfused transcardially with PBS (150 mol/L NaCl, 3 mmol/L KCl, and 5 nmol/L phosphate; pH 7.4, 4 °C). The brains were removed quickly, and sections 1 mm thick were obtained with a cryostat at −7 ± 1 °C. The RVLM was defined according to a rat brain atlas as described previously [3,6,7], and obtained by a punch-out technique. The RVLM tissues were homogenized in 1.15% KCl (pH 7.4) and 0.4% sodium dodecyl sulfate, 7.5% acetic acid adjusted to pH 3.5 with NaOH. Thiobarbituric acid (0.3%) was added to the homogenate. The mixture was maintained at 5 °C for 60 min, followed by heating to 100 °C for 60 min. After cooling, the mixture was extracted with distilled water and *n*-butanolpyridine (15:1) and centrifuged at 1600× *g* for 10 min. The absorbance of the organic phase was measured at 532 nm. The amount of TBARS was determined as oxidative stress by absorbance [3,6,7]. We had previously reported that TBARS is available as an indicator of oxidative stress in the RVLM, despite being an indirect marker [3,6,7]. 

### 4.4. Assessment of Volume Tolerance

At the end of the experiments, the rats that were anesthetized by intraperitoneal injection (2 mL/kg) of a mixture of urethane (250 mg/mL) and α-chloralose (40 mg/mL) and continuous intravenous infusion of the anesthetics at a rate of 0.1 mL/kg/h using a syringe pump (CFV-3200; Nihon Kohden, Tokyo, Japan) were intubated and ventilated artificially with room air (SN-480-7; Shinano, Tokyo, Japan). We infused dextran stepwise, every minute, with a volume of 1–4 mL/kg, and measured left atrial pressure in response to the infused volume, as in our previous study, until the left atrial pressure reached 14–16 mmHg [8].

### 4.5. Statistical Analysis 

Normally distributed variables are expressed as mean ± standard deviation. Data were also analyzed by a 2-factor repeated-measures analysis of variances with Turkey-Kramer post-hoc test. Differences were statistically significant at *p* < 0.05.

## 5. Conclusions

**Figure 8:**
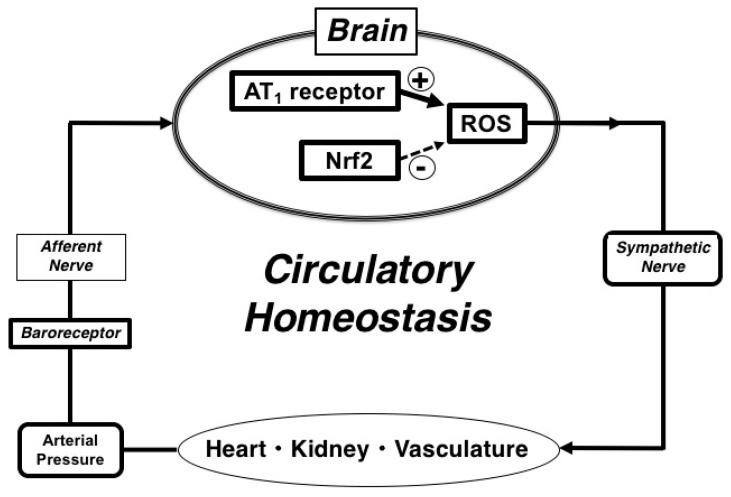
The schema of the concept demonstrated in the present study. + arrow; activation, – dashed arrow; inhibition, AT_1_; angiotensin II type 1, ROS; reactive oxygen species, Nrf2; nuclear factor erythroid 2-related factor 2.

In the present study, we demonstrated that disruption of central antioxidant property of Nrf2 worsened circulatory homeostasis with baroreflex dysfunction in heart failure (Figure 8). Brain renin-angiotensin system and oxidative stress have already been established to induce sympathoexcitation in heart failure. Our results strongly suggest that excess oxidative stress via AT_1_R and impaired antioxidant property with Nrf2 should worsen baroreflex failure and volume tolerance in MI-induced heart failure. We propose that brain AT_1_R and antioxidant Nrf2 could be the novel therapeutic targets to improve circulatory homeostasis via the amelioration of baroreflex and volume tolerance in heart failure.
